# Cognitive consequences of early versus late antiepileptic drug withdrawal after pediatric epilepsy surgery, the TimeToStop (TTS) trial: study protocol for a randomized controlled trial

**DOI:** 10.1186/s13063-015-0989-2

**Published:** 2015-10-26

**Authors:** Kim Boshuisen, Herm J. Lamberink, Monique MJ van Schooneveld, J. Helen Cross, Alexis Arzimanoglou, Ingeborg van der Tweel, Karin Geleijns, Cuno SPM Uiterwaal, Kees PJ Braun

**Affiliations:** Department of Child Neurology, Brain Center Rudolf Magnus, University Medical Center Utrecht, Utrecht, The Netherlands; Department of Child Neuropsychology, Wilhelmina Children’s Hospital, University Medical Center Utrecht, Utrecht, The Netherlands; Neurosciences Unit, UCL-Institute of Child Health, Great Ormond Street Hospital for Children NHS Foundation Trust, London & Young Epilepsy Lingfield, UK; Epilepsy, Sleep and Pediatric Neurophysiology Department (ESEFNP), University Hospitals of Lyon (HCL), Lyon, France; Lyon Neuroscience Research Center, DYCOG team (CRNL, INSERM/CNRS), Lyon, France; Julius Center for Health Sciences and Primary Care, University Medical Center Utrecht, Utrecht, The Netherlands

**Keywords:** Pediatric epilepsy surgery, Antiepileptic drugs, Cognition, Seizure outcome, Behavior, Quality of life

## Abstract

**Background:**

The goals of intentional curative pediatric epilepsy surgery are to achieve seizure-freedom and antiepileptic drug (AED) freedom. Retrospective cohort studies have indicated that early postoperative AED withdrawal unmasks incomplete surgical success and AED dependency sooner, but not at the cost of long-term seizure outcome. Moreover, AED withdrawal seemed to improve cognitive outcome. A randomized trial is needed to confirm these findings. We hypothesized that early AED withdrawal in children is not only safe, but also beneficial with respect to cognitive functioning.

**Design:**

This is a multi-center pragmatic randomized clinical trial to investigate whether early AED withdrawal improves cognitive function, in terms of attention, executive function and intelligence, quality of life and behavior, and to confirm safety in terms of eventual seizure freedom, seizure recurrences and “seizure and AED freedom.” Patients will be randomly allocated in parallel groups (1:1) to either early or late AED withdrawal. Randomization will be concealed and stratified for preoperative IQ and medical center. In the early withdrawal arm reduction of AEDs will start 4 months after surgery, while in the late withdrawal arm reduction starts 12 months after surgery, with intended complete cessation of drugs after 12 and 20 months respectively. Cognitive outcome measurements will be performed preoperatively, and at 1 and 2 years following surgery, and consist of assessment of attention and executive functioning using the EpiTrack Junior test and intelligence expressed as IQ (Wechsler Intelligence Scales). Seizure outcomes will be assessed at 24 months after surgery, and at 20 months following start of AED reduction. We aim to randomize 180 patients who underwent anticipated curative epilepsy surgery below 16 years of age, were able to perform the EpiTrack Junior test preoperatively, and have no predictors of poor postoperative seizure prognosis (multifocal magnetic resonance imaging (MRI) abnormalities, incomplete resection of the lesion, epileptic postoperative electroencephalogram (EEG) abnormalities, or more than three AEDs at the time of surgery).

**Discussion:**

Growing experience with epilepsy surgery has changed the view towards postoperative medication policy. In a European collaboration, we designed a multi-center pragmatic randomized clinical trial comparing early with late AED withdrawal to investigate benefits and safety of early AED withdrawal. The TTS trial is supported by the Dutch Epilepsy Fund (NL 08-10) ISRCTN88423240/ 08/05/2013.

## Background

For children with refractory epilepsy, epilepsy surgery is a successful and widely accepted therapeutic option with postoperative seizure freedom rates ranging from 41 to 93 % [1, 2]. In patients who have been operated on and who have reached seizure freedom the ultimate goal is to discontinue antiepileptic drug (AED) use and to improve developmental capacities [3–5]. AEDs are known to have cognitive side-effects, particularly in children. Most affected cognitive functions are attention, vigilance and psychomotor speed [6–9]. AED reduction has been reported to improve cognitive processing under time pressure [8, 10], psychomotor speed and alertness [11–13], processing speed [14], verbal memory [15] and Intelligence Quotient (IQ) [9]. The growing body of evidence that cognitive functioning improves after AED withdrawal has increased awareness of the possible benefits of the earliest possible withdrawal of AEDs after epilepsy surgery. Two recently published studies [16, 17] showed that early withdrawal of drug treatment unmasks incomplete surgical success and AED dependency sooner, but not at the cost of worse long-term seizure outcome. Thus, early AED withdrawal would discern the few that require further AEDs from the many that can safely stop, without changing the chance of regaining seizure freedom. A pressing question is whether early AED withdrawal improves cognitive outcome measures, as compared to current care with usually later AED withdrawal. To answer that question we designed a pragmatic randomized clinical trial to investigate the benefits and safety of early AED withdrawal after epilepsy surgery in children. We hypothesize that 1) children who discontinue AED’s early have better cognitive scores, behavior and quality of life than those who discontinue late, particularly at 1 year after surgery (when 1 group is without AEDs and the other is still on AEDs), and maybe persisting from then on, and 2) early AED withdrawal is safe and does not cause more recurrences that are unresponsive to restart of medication, than late withdrawal.

### Study objectives

The primary objective is to investigate whether early AED withdrawal improves cognitive function, in terms of attention and executive functions, compared to late AED reduction. The secondary objectives are to assess (improvement in) IQ, and safety, in terms of seizure recurrences, long-term seizure freedom and “seizure and AED freedom” of early versus late AED withdrawal. Seizure freedom will be defined as complete seizure freedom (including auras), expressed as Engel 1A or ILAE class 1 for at least 1 year [18, 19], and “seizure and AED freedom” as being seizure free without medication for at least 1 year. In addition, we will compare behavior, quality of life and other neuropsychological outcomes between the two AED withdrawal strategies.

## Methods/Design

### Study design

The TimeToStop trial will be a European multicenter pragmatic randomized clinical trial. In the Netherlands the trial has been approved by the Medical Ethical Committee of the University Medical Center Utrecht, which is the leading ethical committee. Other participating centers are in the process of protocol submission and have not yet obtained definite approval. Only then, local patient recruitment will start. For every patient who will be included in the trial we will obtain informed consent. In the TimeToStop trial, index intervention is early withdrawal: AED withdrawal is started 4 months after surgery and completed within 8 months after start of withdrawal (thus, at latest 12 months postoperatively). Reference intervention is late AED withdrawal: tapering off medication starts at 12 months after surgery and completion should be within 20 months after surgery. For both treatment arms, the total tapering period is set at 8 months maximum and clinicians may decide which AED they want to taper first and at what speed they will reduce each individual drug. A tapering period of 8 months is considered reasonable, as from the retrospective TimeToStop (TTS) study [16] it could be calculated that patients who fulfill the inclusion criteria used on average 1.7 AEDs prior to surgery (range 0–3). The late (reference) starting point of AED tapering – i.e. 12 months – is based on current practice; the median interval between surgery and drug reduction in our retrospective European study was 12.5 months [16]. The 4 months’ time-point in the early withdrawal group is selected mainly because of logistical reasons. First, informing parents and recruiting patients needs time. Second, inclusion requires proof of early surgical success, which needs a certain time window to discriminate between acute postoperative (running-down) seizures and true seizure recurrence. Third, in some children clinicians may want to prove completeness of resection by performing a postoperative magnetic resonance imaging (MRI), for which a minimum follow-up time of a few months is required. For both intervention strategies, the treating physician is allowed to start tapering earlier if patients experience unacceptable side effects of prescribed AEDs. Rescue medication that is started preoperatively and withdrawn shortly after surgery, can be discontinued according to existing local routine practice, and will not be investigated in this study.

**Fig. 1:**
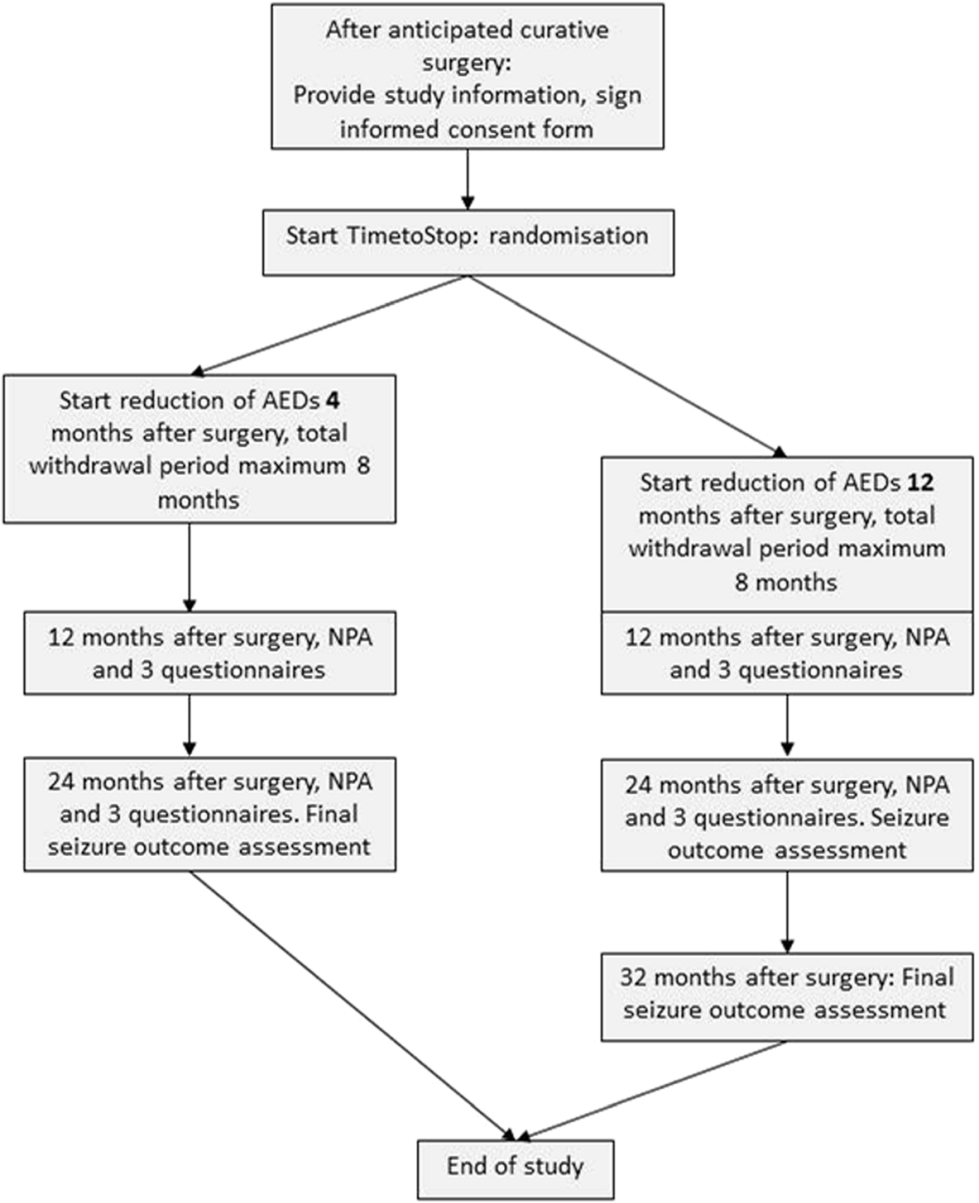
Trial design. Flowchart of trial design; NPA, neuropsychological assessment

The trial’s primary outcome measure at 1 year after surgery is cognitive functioning, in terms of attention and executive functioning, which will be assessed using the EpiTrack Junior [20]. As secondary cognitive outcome measure, intelligence will be tested with the Wechsler Preschool and Primary Scales, the Wechsler Intelligence Scale for children, or the Wechsler Adult Intelligence scale, depending on the children’s age and capabilities; scores derived are Verbal (VIQ), Performal (PIQ) and Full-scale IQ, perceptual organization or reasoning and information processing speed index scores. We will measure baseline neuropsychological status maximum 3 months before surgery (t1), and thereafter at 12 months (±2 months) (t2) and 24 months (±2 months) (t3) postoperatively. Patients and/or parents will at all neuropsychological test points complete three types of questionnaires to assess the secondary outcome measures: the Child Behavior Checklist (CBCL) and the the Hague Side Effects Scale (HASES) [21] will be completed by the parents, and the Pediatric Quality of Life Inventory^TM^ (PedsQL™) will be completed by both parents and patients. This design will allow us to assess whether cognitive performance is better in the early AED-withdrawal group, with patients being AED-free at 1 year following surgery, compared to the group that is still on full medication at the first postoperative time point. It will also enable the study of differences at a later follow-up time point, when both groups have completely discontinued medication (t3) (Fig. 1). Seizure outcome will be assessed at the moment of randomization, as seizure freedom is an inclusion criterion, at 24 months after surgery, and at 20 months following start of AED withdrawal. This design allows us to assess differences in seizure outcome at equal AED-free intervals for both groups, and at 2 years after surgery irrespective of length of AED freedom.

Patients will be allocated to treatment strategy by concealed blocked randomization and randomization will be stratified for preoperative IQ score and medical center. The primary goal of randomization is to achieve an AED treatment duration contrast. Therefore, physicians and patients allocated to an arm will be instructed to comply with that strategy. However, these intervention strategies will to a certain extent be in compliance with clinical practice, and allow “protocol violations” for the following instances: if patients have seizure recurrences in the period before scheduled AED withdrawal, they do not have to start AED withdrawal. The same applies to seizure recurrences that occur after start of tapering medication. Patients can restart medication and do not have to taper medication again, regardless of planned medication status. Notably, whatever “protocol deviations” will occur, patients will be analyzed as randomized (intention-to-treat principle).

### Inclusion criteria

In order to be eligible a subject must meet all of the following criteria:

- Younger than 16 years at surgery, with focal non-idiopathic epilepsy
- Native speaker in the language in which the neuropsychological tests have to be taken
- Able to perform an EpiTrack Junior [20] test preoperatively
- Underwent intentionally curative epilepsy surgery
- After surgery, the treating physician considers withdrawal of AEDs, with the intention to completely discontinue medication
- The treating physician, the patient (if capable), and the parents agree with randomization in either arm of the study
- Postoperative seizure freedom was achieved (with the exception of so-called running down seizures not outlasting 2 weeks)
- Written informed consent of children and both parents or caregivers of children older than 12 years, and of both parents or caregivers of children below that age

### Exclusion criteria

A subject who meets any of the following criteria will be excluded from participation:

- A contraindication to be randomized to either of the two withdrawal strategies
- The treating physician does not want to discontinue all AEDs within a maximum time frame of 8 months as prescribed in the study protocol
- Multifocal MRI abnormalities, known incomplete resection of the anatomical or epileptogenic lesion certified before randomization (if considered necessary by the treating physician a postoperative MRI may be performed) and, if a postoperative electroencephalogram (EEG) is performed before randomization (at the discretion of the treating physician), epileptic EEG abnormalities. These are the most important risk factors of seizure recurrence or unfavorable long-term seizure outcome [16]
- Use of more than three AEDs at the time of surgery. The reason is that clinicians can then not be expected to want to wait for 12 months in the late withdrawal arm to reduce the first AED in these patients. Furthermore, withdrawing 4 or more AEDs within 8 months may be difficult to achieve
- Patients who are on a ketogenic diet or have a vagal nerve stimulator implanted
- If surgery is primarily intended as “tumor surgery” (a growing epileptogenic lesion was the indication for surgery) and not as epilepsy surgery

#### Excluded patients

Participating centers will provide brief anonymized information about all children who are operated on, including those who were not in the trial, to document reasons for exclusion and the overall proportion of eligible patients, allowing the assessment of generalizability of the trial results. We will only report aggregated data of excluded patients.

### Statistical analysis

The general approach towards analysis will be on an intention-to-treat basis, so patients randomized will be analyzed and accounted for as allocated, independent of later compliance.

For all analyses described below, we will add baseline adjusted analyses using various regression techniques (linear regression for continuous and logistic regression for binary outcomes). For these adjustments to increase statistical precision, we will use propensity score methods or inverse variance weighting methods.

Prior to analyses described below, we will deal with missing values by formal accepted multiple imputation methods, for reasons of optimizing statistical precision but more importantly because particularly loss to follow-up may be expected not to occur randomly in this trial.

#### Outcome parameters

Cognitive outcome: differences in EpiTrack scores, IQ scores and other continuous outcome measures of neuropsychological assessment will be analyzed using independent samples *t*-tests or other tests depending on distributions. EpiTrack and IQ differences will be assessed cross-sectionally to answer the primary research question. Additionally, longitudinal changes in EpiTrack measures and IQ compared to preoperatively will be assessed between both treatment arms using linear mixed models. We will calculate a number needed to treat, expressed as the number of patients required to taper drugs earlier to achieve one more patient with improvement in attention. Analyses will be considered statistically significant if 95 % confidence intervals for group differences do not include 0, compatible with a 2-sided *p* < 0.05. For chances of eventual seizure freedom and “seizure and AED freedom”; we will calculate a relative risk and a risk difference with 95 % confidence intervals. Chances of eventual seizure freedom and “seizure and AED freedom” will be compared between the groups at 24 months after surgery and at 20 months following start of AED withdrawal; for the early withdrawal arm this will be at 24 months and for the late withdrawal arm at 32 months.

### Sample size calculation

The study is powered based on the primary neuropsychological measures attention and executive function, determined with the EpiTrack Junior. We plan to study a continuous response variable from a late withdrawal (control) versus an early withdrawal (experimental) group with one control per experimental subject. For the sample size calculation, we used the reference values given in the Introduction and first validation of EpiTrack Junior [20], which presents reference values of healthy children and children with epilepsy. The mean (SD) EpiTrack Junior score in healthy children was 32.6 (2.40). In children with epilepsy the mean (SD) score was 29.5 (4.70). For sample size calculations, we calculated the sample size using the SD of the epilepsy patients. Based on numbers of patients operated on per year in the participating medical centers and excluding patients with incompletely resected lesions, multifocal MRI abnormalities, an epileptic EEG postoperatively and use of a maximum of three AEDs at time of surgery (the numbers derived from the previous retrospective study [16]), we estimated to expect to include at least 90 patients per year. By including at least 150 patients in the trial, with 75 patients per arm, we will be able to detect a true difference in the mean response of 2.16 points in EpiTrack Junior score (which corresponds to a difference of 0.46 SD) with a 2-sided alpha = 0.05 and power (1-beta) = 0.8. This difference is considered clinically relevant, as for the EpiTrack a 0.5 SD difference, can reflect a shift towards a milder impaired group of epilepsy patients [20]. One interim analysis according to O’Brien Fleming is planned after half of the planned number of patients has reached their endpoints. For reasons of possible dropout, we will aim to include 180 patients.

### Ethical approval

The multicenter trial will start in the University Medical Center Utrecht, the Netherlands, in November 2015. Ethical permission has been obtained from the ethical committee of the University Medical Center Utrecht, which is the leading ethical committee. Eight sites: University Hospitals of Lyon (HCL), France; University Hospital Strasbourg, France; Epilepsy Center Freiburg, Epilepsy Center Kork, University Hospital Heidelberg, Germany; Great Ormond Street Hospital for Children NHS Foundation Trust, London, and Young Epilepsy, Lingfield, United Kingdom; Western General Hospital Edinburgh, Scotland; Hôpitaux Universitaire de Genève, Switzerland, will join the trial shortly thereafter. Other centers, that have adequate experience with pediatric epilepsy surgery, are welcome to participate.

### Safety monitoring

To minimize the risk for the participants in the proposed trial, we will exclude patients with those characteristics that have previously been shown to predict unfavorable outcome [16]. In our retrospective cohort we simulated the prospective randomized trial on 478 patients who would be eligible based on their exact inclusion and exclusion criteria. In this group, the interval to start of AED reduction was not associated with seizure recurrences during or after AED withdrawal (*p* = 0.155, HR 0.94; CI 0.86–1.02 per 3 months), nor with eventual seizure freedom (*p* = 0.551, HR 0.96; CI 0.85–1.09) or seizure and AED freedom (*p* = 0.516, HR 1.01 CI 0.97–1.06, Cox regression analysis). Based on these analyses, we expect an overall comparable percentage of seizure recurrences in both treatments arms, similar to the number or recurrences encountered in everyday clinical practice when starting tapering of AEDs.

We appointed a Data Safety Monitoring Board (DSMB) that will analyze our data to warrant safety and to monitor inclusion rates and assess inclusion feasibility. At 24 months a blinded interim-analysis will be performed by the DSMB to investigate superiority of one of the treatment arms with regard to the primary outcome measure. If the difference in attention between groups is more than 1 SD (age appropriate average reaction time), the DSMB may advise to prematurely stop the trial, if early withdrawal shows to be superior, the remaining patients in the late withdrawal group who still await start of AED reduction are allowed to withdraw medication from that time on. A test result with a 2-sided *p* < 0.0054 will be declared significant.

**Fig. 2:**
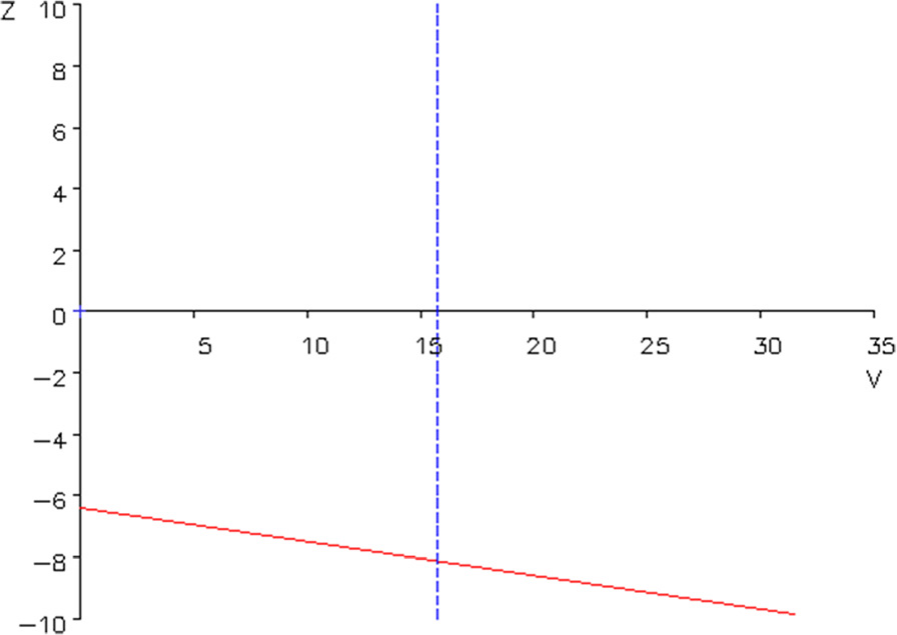
Sequential testing safety profile. The plot is based on a likely sample size of 75 patients per treatment strategy, a 1-sided α = 0.05, an expected seizure recurrence rate ≤ 30 % and a cut-off point for safety, which is a recurrence rate of ≥ 40 %. The red boundary represents the safety margin. The following formulas will be used to plot data in the graph: *Ζ* = −(*F* − *n* × *Pc*) (→*Ζ* = number of observed seizure recurrences – number of expected seizure recurrences), *V* = *n* × *Pc* (1 − *Pc*) (→*V* is the variance of *Z* and stands for the amount of information in *n* patients, with *n* being the number of included patients, *Pc* the expected seizure recurrence risk (i.e., for the selected patient cohort: 0.30) and *F* the number of observed seizure recurrences). The vertical line is the anticipated value for *V* for a sample size of 75 patients

With regard to safety; due to the design of the trial, an interim safety analysis comparing both treatment arms at one moment is not possible. This is because we expect in both arms relapse risks to increase particularly once AED withdrawal is started. Thus, in the early withdrawal group recurrences probably occur sooner than in the late withdrawal group, although the total risk of relapse at the end of follow-up is not expected to differ between groups. This implies that at interim time points, when inherently more patients are under AED reduction (at increased risk of relapse) in the early arm, a fair comparison of relapse between both groups is not feasible. To deal with this, we will assess safety in each arm separately. The study intervention will be considered safe when seizure recurrences occur in less than 40 % of both treatment arms. This percentage is chosen based on the general seizure freedom rate after childhood epilepsy surgery of 41 to 93 % [1, 2]. Based on previous identification of risk factors for seizure recurrences, we expect recurrence rates in the patients included in the trial to be < 30 %, due to exclusion of patients with identified risk factors [1, 16, 22, 23]. We do not expect, however, the seizure status to be better than in normal clinical practice. Because of the expected cognitive benefit due to a longer AED-free period, a 40 % rate of seizure relapse will still be accepted. The DSMB statistician will analyze the number of patients with seizure recurrences every 3 months during trial duration, in order to allow early awareness of unexpected high numbers of recurrences, exceeding the 40 % limit. In this sequential testing safety model (Fig. 2), a 1-sided *p* value < 0.05 will be considered significant for the safety monitoring [24, 25].

### Adverse event reporting

For this study, recurrent epileptic seizures during AED withdrawal that need hospitalization do not necessarily have to be recorded as serious adverse events (SAEs), but are noted in the case report form (CRF) as one of the outcome measures, and will be included in the interim safety analyses by the DSMB. Epileptic seizures that lead to a potentially life-threatening situation, such as status epilepticus, will be reported as SAEs.

All SAEs will be reported through the web portal “ToetsingOnline,” to the accredited ethical committee that approved the protocol, within 15 days after the sponsor has first knowledge of the serious adverse reactions. Participating centers will inform the sponsor about the SAEs. Reporting of SAEs that result in death or are life-threatening should be expedited. The expedited reporting will occur not later than 7 days after the responsible investigator has first knowledge of the adverse reaction. This is for a preliminary report with another 8 days for completion of the report.

## Discussion

We present the protocol of a European pragmatic randomized clinical trial, designed to investigate the possible benefits and the safety of early AED withdrawal after epilepsy surgery in children. We hypothesize that early AED withdrawal is safe, in terms of seizure relapse rates and eventual seizure outcomes, and will improve cognitive functioning, behavior and quality of life, compared to late withdrawal. We will investigate these hypotheses by randomly allocating patients to an early withdrawal group that starts withdrawal of medication 4 months after surgery, versus a late withdrawal group, that starts withdrawal of medication after 12 months. Justification of this trial needs to be discussed. The non-experimental evidence obtained so far on safety of AED withdrawal in relation to seizure outcome seems strong and consistent [16, 17]. In analyses that had been elaborately adjusted for confounding, there was a slightly increased recurrence risk for earlier withdrawal, but not for regain of seizure freedom rates and eventual seizure outcome. Moreover, findings on cognitive outcome after AED withdrawal all pointed towards cognitive improvement on several domains [3–15]. Why then do we think that the trial will have important added value? Through randomized allocation the study population will be broader than in the non-experimental studies which only pertained to patients for whom AED withdrawal had already been decided about. Consequently, results will have wider clinical applicability and will apply to all patients who are eligible for withdrawal decision- making. A very important asset will be obtaining baseline comparability of treatment arms for both known and measurable confounders, but also for unknown or unmeasurable confounders. In principle, the non-experimental evidence, although elaborately adjusted for known confounders, does leave residual confounding as an explanation. The trial will allow for full follow-up of all children randomized and for intention-to-treat analysis. Finally, through the above, stronger evidence will contribute to our ultimate goal, implementation of evidence-based practice concerning AED withdrawal. Although previous non-experimental research does seem to indicate benefits of early AED withdrawal, it is especially the remaining uncertainty about residual confounding that leads us to claim that there currently is genuine equipoise about timing of withdrawal and, therefore, that random allocation is ethically justified. This issue will have to be addressed with regard to trial feasibility. Throughout European centers participating in the trial, there are regional preferences concerning AED withdrawal timing, and non-experimental evidence has already led to changes in clinical practice concerning withdrawal timing. It will be vital for successful completion of the trial that participating physicians can and will be convinced that there still is true equipoise, as a sufficient basis for studying randomized AED withdrawal timing to the ultimate benefit of children who have undergone epilepsy surgery.

Recruitment of participants for this study might be challenging for three reasons. First, in some of the participating countries and centers, AED withdrawal policies are still rather conservative, conflicting with the proposed early AED strategy. This may either be due to reluctance of parents or patients to risk a seizure relapse, now that seizure freedom has finally been achieved following epilepsy surgery, or to fear of the treating neurologist or neurosurgeon that seizure control is permanently lost after a post-withdrawal relapse. In addition, in some patients treating physicians may require a postoperative MRI to prove complete resection of the epileptogenic lesion before considering AED withdrawal. Planning of MRI scanning before randomization may not always be feasible in the proposed short time period. Second, in many participating centers, AED withdrawal policies already tend to change towards earlier reduction, as a consequence of the findings from the previous retrospective observational TTS study [16]. These changing opinions on AED withdrawal policies are not only known among physicians, there is also an increasing awareness of safety of early withdrawal among parents of children who have been operated on. Therefore, willingness to be randomized to the late withdrawal strategy arm may prove difficult in these centers. Third, some treating physicians may not want to withdraw AEDs within a set maximum time frame of 8 months, or only consider reduction of medication instead of complete discontinuation of AEDs, fearing that relatively early, rapid or complete withdrawal may increase the risk of seizure relapse. To prevent limited recruitment of patients we will encourage participating physicians to counsel parents and patients optimally, based on the available evidence regarding safety of AED withdrawal, which can be summarized as follows: although retrospective data suggest that early withdrawal is safe and does not compromise eventual seizure outcome or treatability of seizure relapse, definite proof of safety in a predefined population of children who underwent anticipated curative surgery is still required, justifying randomization to either treatment arm. Furthermore, although we anticipate cognitive improvement following AED discontinuation, the – possibly enduring – cognitive advantages of early over late withdrawal need to established, which is important to enable careful balancing of risks and benefits of early AED discontinuation in individual patients in the future.

### Trial organization

#### Steering committee

The steering committee carries the ultimate responsibility for the trial. Specific tasks of the steering committee are:

1. Design and final approval of the study protocol
2. Approval of the amendments to the study protocol
3. Deciding whether or not to continue the trial based on the recommendations of the DSMB
4. Approval of manuscripts and publications of the trial

The Trial committee is constituted of the principle investigator of each participating center and of the members of the Steering committee.

As of 16 March 2015, the members of the Steering Committee are (in alphabetical order): A. Arzimanoglou, child neurologist, University Hospitals of Lyon, France, co-principle investigator; K. Boshuisen, research physician, University Medical Center Utrecht, the Netherlands; K.P.J Braun, child neurologist, University Medical Center Utrecht, the Netherlands, co-principle investigator; J.H. Cross, child neurologist, Great Ormond Street Hospital for Children NHS Foundation Trust, London, and Young Epilepsy, Lingfield, United Kingdom, co-principle investigator; K. Geleijns, child neurologist University Medical Center Utrecht, the Netherlands, coordinating investigator; H.J. Lamberink, PhD student, University Medical Center Utrecht, the Netherlands; M.M.J. Schooneveld, child neuropsychologist, University Medical Center Utrecht, the Netherlands; C.S.P.M Uiterwaal, epidemiologist and statistician, University Medical Center Utrecht, the Netherlands.

Principle and coordinating investigators (other than those listed in the steering committee) at each site are in alphabetical order: R. Chin, child neurologist, Western General Hospital Edinburgh, Scotland; T. Polster, child neurologist, Krankenhaus Mara, Epilepsiezentrum Bethel, Bielefeld, Germany; G. Ramantani, child neurologist, Epilepsy Center Freiburg, Epilepsy Center Kork, University Hospital Heidelberg, Germany; A. De Saint-Martin, child neurologist, University Hospital Strasbourg, France; M. Seeck, epileptologist, Hôpitaux Universitaire de Genève.

### Data Safety Monitoring Board

The DMSC analyses the unblinded data on a permanent basis and formulates recommendations for the Steering Committee on the continuation of the trial. The Data Monitoring Committee may also offer unsolicited recommendations. Members of the Data Monitoring Committee are: Prof. Dr. Oebo Brouwer, child neurologist, epileptologist, UMCG; Dr. Ingeborg van der Tweel, statistician, UMCU; Prof. Dr. Jaap Kappelle, neurologist, UMCU.

## Trial status

Ethical approval has been obtained for the University Medical Center Utrecht. The other participating centers are in the process of obtaining approval. No patients have yet been included in the trial.

## Abbreviations

AED: antiepileptic drug
CBCL: Child Behavior Checklist
CI: confidence interval
CRF: case report form
DSMB: Data Safety Monitoring Board
EEG: electroencephalogram
HASES: The Hague Side Effects Scale
HR: hazard ratio
IQ: Intelligence Quotient
MRI: magnetic resonance imaging
PedsQL^TM^: Paediatric Quality of Life Inventory^TM^
SD: standard deviation
PIQ: Performal IQ
SAE: severe adverse event
SD: standard deviation
TTS: TimeToStop
VIQ: Verbal IQ
